# A Novel Technique of Robotic Buccal Mucosal Graft Ureteroplasty for Proximal Ureteric Strictures by Non-transecting Mucosa-to-Mucosa Anastomosis

**DOI:** 10.7759/cureus.86387

**Published:** 2025-06-19

**Authors:** Mukkani Velan, Venkata Krishnan S, Ajay Ramesh, Nitesh Jain, Sandeep Bafna

**Affiliations:** 1 Urology, Apollo Hospitals, Chennai, IND; 2 General Surgery, Saveetha Medical College and Hospital, Chennai, IND

**Keywords:** msanta ureteroplasty, mucosa sparing ureteroplasty, non-transecting ureteroplasty, robotic buccal ureteroplasty, upper ureteric strictures

## Abstract

Buccal mucosal graft (BMG) ureteroplasty is a well-established technique for complex upper ureteric strictures, particularly with robotic assistance. However, limited guidance exists for cases involving a narrow ureteral plate. We present a novel technique employing a non-transecting mucosa-to-mucosa anastomosis at the stricture site, followed by BMG augmentation. This method adapts the principles of non-transecting urethroplasty (mucosal-sparing augmented non-transected anastomotic (MsANTA)) to the ureter, aiming to preserve vascularity. Patients underwent preoperative CT urography and intraoperative retrograde studies to delineate the stricture. After harvesting the buccal graft and identifying the narrow ureteral plate, mucosal approximation without transection was performed to widen the lumen, followed by watertight graft anastomosis. This vascular-preserving modification may reduce stricture recurrence. Further comparative studies are warranted to validate its efficacy.

## Introduction

The incidence of benign upper ureteric strictures has increased due to the widespread use of endourological interventions and iatrogenic causes, especially following ureteroscopy for urolithiasis [1,2]. These strictures are often difficult to treat and require prompt management to preserve renal function.

Robotic-assisted buccal mucosal graft (BMG) ureteroplasty has become a preferred option for long-segment and complex upper ureteric strictures. The main principles of ureteral reconstruction include achieving a watertight lumen restoration while preserving the ureteral vascularity. This may be accomplished through primary anastomosis, flaps, or grafts [3]. However, traditional techniques involving ureteral transection may compromise the segmental vascularity of the ureter.

Our novel technique utilizes a non-transecting mucosa-to-mucosa anastomosis at the strictured segment, followed by buccal mucosal graft augmentation. This approach preserves vascularity while restoring the luminal patency.

## Technical report

Patient selection and evaluation

**Figure 1:**
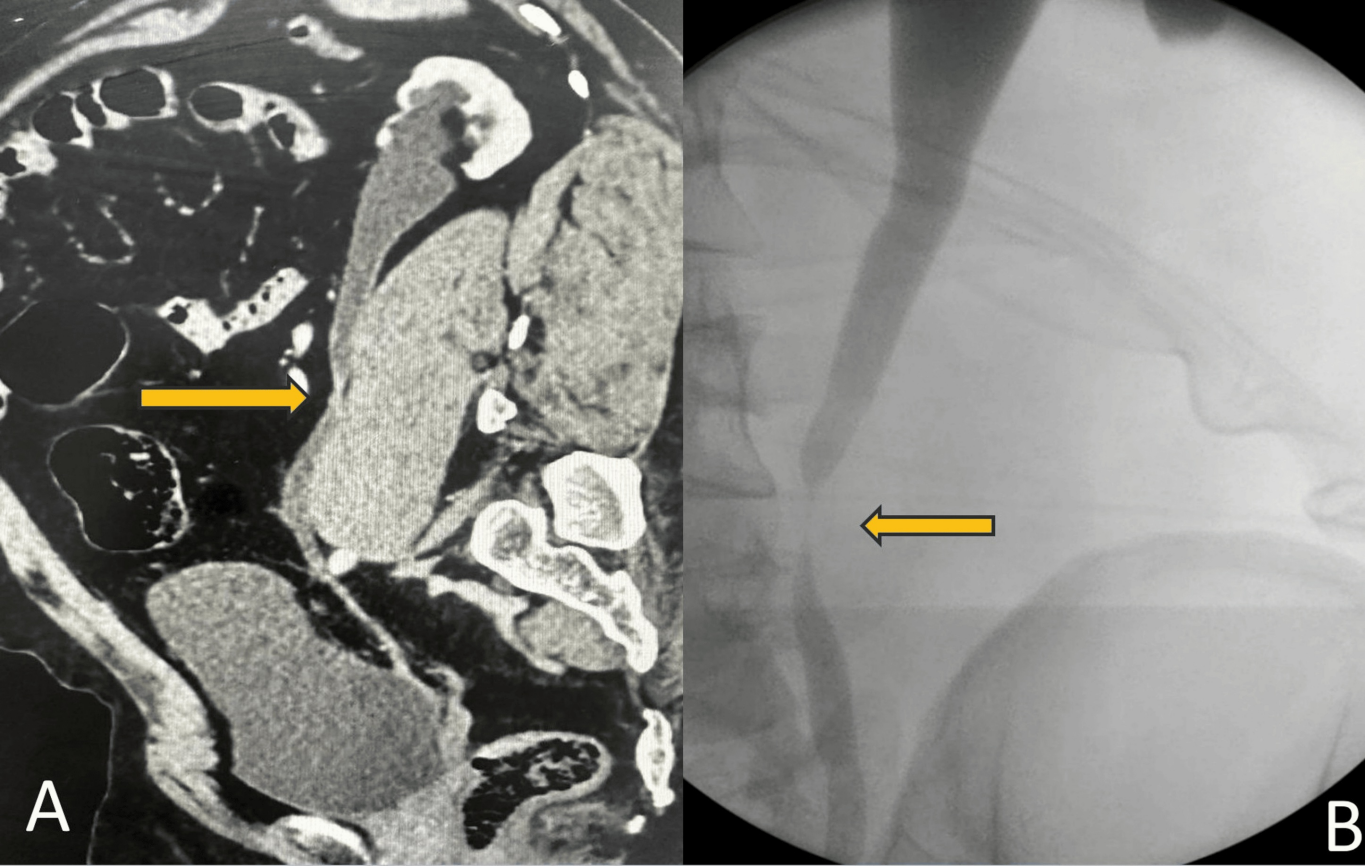
(A) Computed tomography image showing upper ureteric stricture in sagittal view; (B) retrograde pyelogram confirming the stricture site.

All patients underwent thorough evaluation, including history taking, clinical examination, serum biochemistry, CT intravenous urography (CT-IVU), and a dimercaptosuccinic acid (DMSA) renal scan. Patients who have stricture on imaging with preserved renal function are selected for surgery. CT-IVU and intraoperative retrograde pyelography were used to determine the location and extent of the ureteral stricture (Figure 1).

Graft harvesting

**Figure 2:**
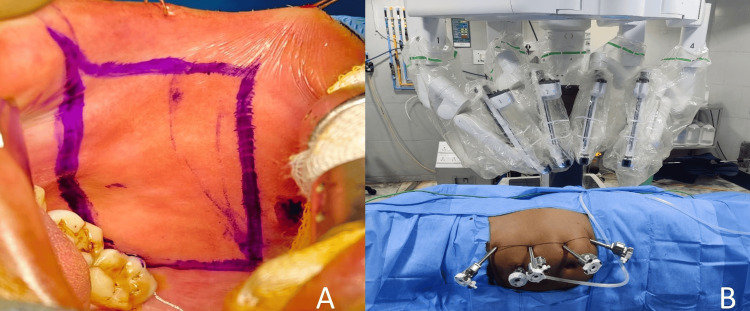
(A) Marking for buccal graft; (B) patient positioning for robotic ureteroplasty.

Under general anesthesia (with transnasal intubation), the retrograde study was done to delineate the stricture, and a 5 Fr ureteric catheter (Biorad Medisys, Pune, India) was placed retrogradely up to the stricture site. Once the length of the stricture site was determined, the buccal mucosal graft was harvested prior to docking the robot from either of the cheeks after infiltration with local anesthesia (Figure 2a). Buccal mucosa, being hairless and robust with an excellent vascular lamina propria, is ideal for grafting in urological reconstruction [4]. The graft was taken 1-2 cm longer than the measured stricture. Hemostasis was secured, and the donor site was left to epithelialize. The graft was defatted and soaked in an antibiotic (ampicillin and cloxacillin 1 gm in 100 ml saline) solution.

Robotic setup and ureteroplasty

**Figure 3:**
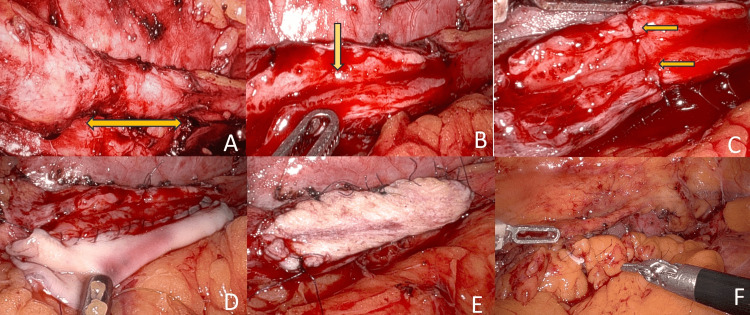
(A) Strictured segment; (B) narrow ureteral plate; (C) widened ureteral plate after mucosa-mucosa anastomosis; (D) and (E) placement of graft and anastomosis; (F) omental wrapping after anastomosis.

The patient was repositioned to a modified flank position. Four 8-mm robotic ports and one 12-mm assistant port were placed (Figure 2b). Following medial mobilization of the colon, the ureter was identified and dissected cranially and caudally around the stricture segment (Figure 3a).

The ureter was incised longitudinally at the stricture site until healthy mucosa was seen on either end. The mucosal plate was evaluated. If narrow, mucosa-to-mucosa approximation using 5-0 polydioxanone (PDS) (Ethicon Inc., Cincinnati, OH) was performed to widen the ureteral plate (Figures 3b, 3c).

**Figure 4:**
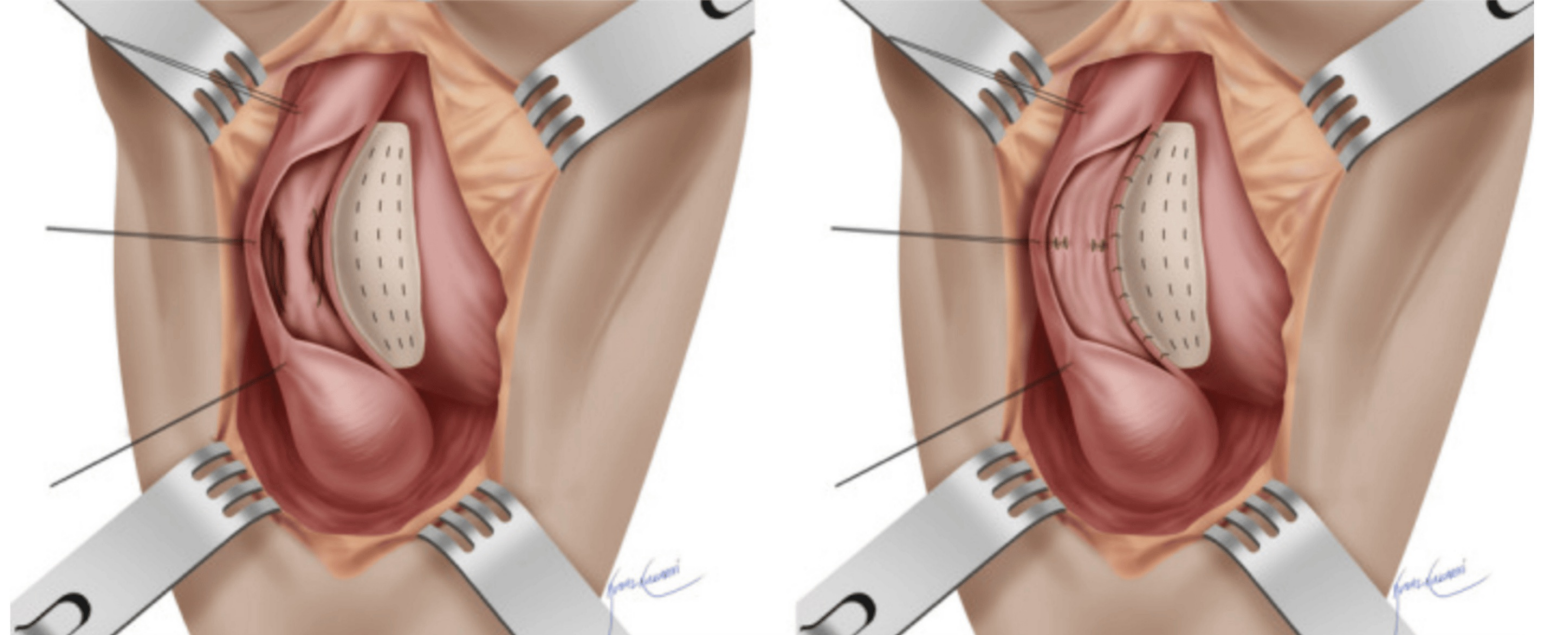
MsANTA urethroplasty-Joshi step. MsANTA: mucosal-sparing augmented non-transected anastomotic. Joshi step [6], where the urethral plate is widened before anastomosis with graft. Image by Joshi et al. 2022 [6]. Reproduced with open access permissions under the terms and conditions of the Creative Commons Attribution (CC BY) license (https://creativecommons.org/licenses/by/4.0/).

The buccal graft was introduced via the assistant port (Figure 3d) and anastomosed to the widened ureteral plate using 4-0 Polyglactin sutures (Ethicon Inc., Cincinnati, OH) in a watertight fashion (Figure 3e). A 5 Fr double-J stent (Biorad Medisys, Pune, India) was inserted before the completion of the anastomosis. The omental wrapping was done to reinforce the repair (Figure 3f). This approach follows the same concept as the "Joshi step" in mucosal-sparing augmented non-transected anastomotic (MsANTA) urethroplasty (Figure 4) [5,6].

Post-operative care

The Foley catheter was routinely removed on post-operative day (POD) six. In the absence of increased drain output, the surgical drain was subsequently removed on POD seven. A double-J ureteral stent was maintained in situ for a duration of six weeks. Following stent removal, patients undergo follow-up imaging with CT urography at three months post-operatively to assess the integrity of the repair and ensure adequate ureteral patency.

## Discussion

Robotic buccal mucosal graft (BMG) ureteroplasty is increasingly recognized as an effective treatment for long and complex upper ureteric strictures, particularly in the context of failed endourological procedures or iatrogenic injuries. Traditional ureteroplasty techniques often involve ureteral transection, which can jeopardize the segmental blood supply and increase the risk of ischemia and recurrent stricture, especially in patients with prior surgery or radiation exposure [7].

In this report, we propose a vascular-preserving, non-transecting technique using mucosa-to-mucosa approximation at the strictured segment followed by BMG augmentation. The concept is adapted from the MsANTA urethroplasty approach described by Joshi et al. [6], which aims to preserve the native tissue architecture and vascularity by avoiding complete transection of the urethra.

This technique is supported by a strong historical and biological foundation. The use of buccal mucosa in urological reconstruction was first described by Humby in 1941 [8] and later standardized by Morey and McAninch in the 1990s for urethral reconstruction [9]. Buccal mucosa has ideal graft characteristics, including a thick, vascular lamina propria and resistance to infection, making it suitable for urinary tract reconstruction [10].

Recent literature supports the move towards non-transecting ureteroplasty. Sahay et al. (2024) reported favorable outcomes with laparoscopic and robotic BMG ureteroplasty, emphasizing the importance of ureteral plate preservation [7]. Similarly, Lee et al. (2021) emphasized that careful graft bed preparation and preservation of the ureteral adventitia are critical to graft success and long-term patency [3]. Ganpule et al. (2018) provided detailed insights into inlay and onlay grafting techniques during robotic BMG ureteroplasty, underlining the importance of meticulous tissue handling and hemostasis [2].

Our technique integrates these principles and advances them by employing mucosal approximation to widen a narrow ureteral plate, followed by watertight graft augmentation. This method minimizes ischemic insult and leverages the advantages of minimally invasive robotic platforms, including enhanced dexterity and precision.

Moreover, the pathological heterogeneity of benign ureteral strictures, as described by Tan et al. (2022), necessitates individualized reconstructive approaches [1]. By adapting the repair to the condition of the ureteral plate, our approach aligns with modern trends in personalized surgical reconstruction.

In sum, this technique represents a conceptual shift toward preserving native tissue and vascularity while ensuring luminal patency. It is a logical progression of urethral reconstructive principles applied to ureteral surgery. With further validation through larger prospective studies, this approach could become a new standard for selected patients with complex upper ureteric strictures.

## Conclusions

Preservation of ureteral vascularity is pivotal in the success of reconstructive procedures for upper ureteric strictures. Our novel, robotic-assisted, non-transecting mucosa-to-mucosa anastomosis with buccal mucosal graft augmentation offers a viable and innovative solution, particularly when a narrow ureteral plate is encountered. By maintaining the ureteral axis and vascularity, this technique holds promise in improving long-term outcomes. With the growing adoption of robotic surgery, such vascular-sparing adaptations are poised to become an integral part of the ureteric reconstructive armamentarium. Larger prospective studies and long-term follow-up will be key to establishing this approach as a new standard.
